# Stat3 as a potential therapeutic target for rheumatoid arthritis

**DOI:** 10.1038/s41598-017-11233-w

**Published:** 2017-09-08

**Authors:** Takatsugu Oike, Yuiko Sato, Tami Kobayashi, Kana Miyamoto, Satoshi Nakamura, Yosuke Kaneko, Shu Kobayashi, Kengo Harato, Hideyuki Saya, Morio Matsumoto, Masaya Nakamura, Yasuo Niki, Takeshi Miyamoto

**Affiliations:** 1 Department of Orthopedic Surgery, Keio University School of Medicine, 35 Shinano-machi, Shinjuku-ku, Tokyo, 160-8582 Japan; 2 Department of Advanced Therapy for Musculoskeletal Disorders, Keio University School of Medicine, 35 Shinano-machi, Shinjuku-ku, Tokyo, 160-8582 Japan; 3 Department of Musculoskeletal Reconstruction and Regeneration Surgery, Keio University School of Medicine, 35 Shinano-machi, Shinjuku-ku, Tokyo, 160-8582 Japan; 4 Division of Gene Regulation, Institute for Advanced Medical Research, Keio University School of Medicine, 35 Shinano-machi, Shinjuku-ku, Tokyo, 160-8582 Japan

## Abstract

Rheumatoid arthritis (RA) is a multi-factorial disease characterized by chronic inflammation and destruction of multiple joints. To date, various biologic treatments for RA such as anti-tumor necrosis factor alpha antibodies have been developed; however, mechanisms underlying RA development remain unclear and targeted therapy for this condition has not been established. Here, we provide evidence that signal transducer and activator of transcription 3 (Stat3) promotes inflammation and joint erosion in a mouse model of arthritis. Stat3 global KO mice show early embryonic lethality; thus, we generated viable Stat3 conditional knockout adult mice and found that they were significantly resistant to collagen-induced arthritis (CIA), the most common RA model, compared with controls. We then used an *in vitro* culture system to screen ninety-six existing drugs to select Stat3 inhibitors and selected five candidate inhibitors. Among them, three significantly inhibited development of arthritis and joint erosion in CIA wild-type mice. These findings suggest that Stat3 inhibitors may serve as promising drugs for RA therapy.

## Introduction

Rheumatoid arthritis (RA), a chronic inflammatory disease, consists of symptoms such as continuous inflammation, swelling, destruction and pain in multiple joints, and is a condition that limits patients’ quality of lives^1^. Various factors including genetic and environmental factors or minor infections are thought to promote RA development^2^; however, pathological mechanisms underlying RA remained unclear. To date, biologics such as tumor necrosis factor alpha (TNFα) blockers^3^ have been used as RA therapy, as have non-steroidal anti-inflammatory drugs (NSAIDs), steroids, and disease-modifying anti-rheumatic drugs (DMARDs) such as methotrexate followed by TNFα inhibitors^4^.

Some report that amplification of IL-6 signaling and/or on-going infections underlie the chronic inflammation seen in RA^5^. Previously, we reported that signal transducer and activator of transcription 3 (Stat3) functioned in a positive feedback loop that drove expression of inflammatory cytokines and receptor activator of nuclear factor kappa B ligand (RANKL) and led to concomitant inflammation and osteoclastogenesis, which is required for joint destruction^6^. However, Stat3 function in RA development has not been assessed *in vivo* in a genetic model, since Stat3 global knockout mice show embryonic lethality.

Stat3 is activated by upstream cytokines, among them IL-6 family factors such as IL-6 and Oncostatin M^7^. Thus, Stat3 reportedly plays an important role in mediating inflammatory signals^8^. Stat3 is also required for embryonic development: Stat3 global knockout (KO) mice exhibit lethality between embryonic days 6.5 and 7.5^9^. As a result, analysis of various Stat3 functions in adults has required establishment of Stat3 conditional KO mice^10–12^.

Drug repositioning enables clinicians to utilize reagents approved to treat other diseases as therapy for a different disease^13, 14^. Since the former have already received approval as human therapies, large clinical trials of safety are unnecessary, saving time and expense. Several agents have been approved for new indications by this method^14^.

Here, we established Stat3 conditional KO in adults by crossing Mx Cre and Stat3-flox mice to yield Mx Cre/*Stat3* ^*flox/flox*^ mice. Stat3 deletion blocked both joint inflammation and destruction in collagen-induced arthritis (CIA) models. Global inhibition of Stat3 in adults did not promote lethality, suggesting that Stat3 can be targeted in adults. We then undertook a screen for reagents to inhibit Stat3 activation using ninety-six existing drugs, identified five candidate inhibitors, and found that three of those blocked arthritis in a CIA model. Among them, meloxicam exhibited the best effects and inhibited serum IL-6 elevation and articular cartilage erosion in that model. Thus, here we have employed an animal model useful to identify Stat3-inhibiting agents and show that Stat3 could potentially serve as a therapeutic target to treat RA.

## Results

### Stat3 loss blocks joint inflammation in a mouse model of arthritis

**Figure 1:**
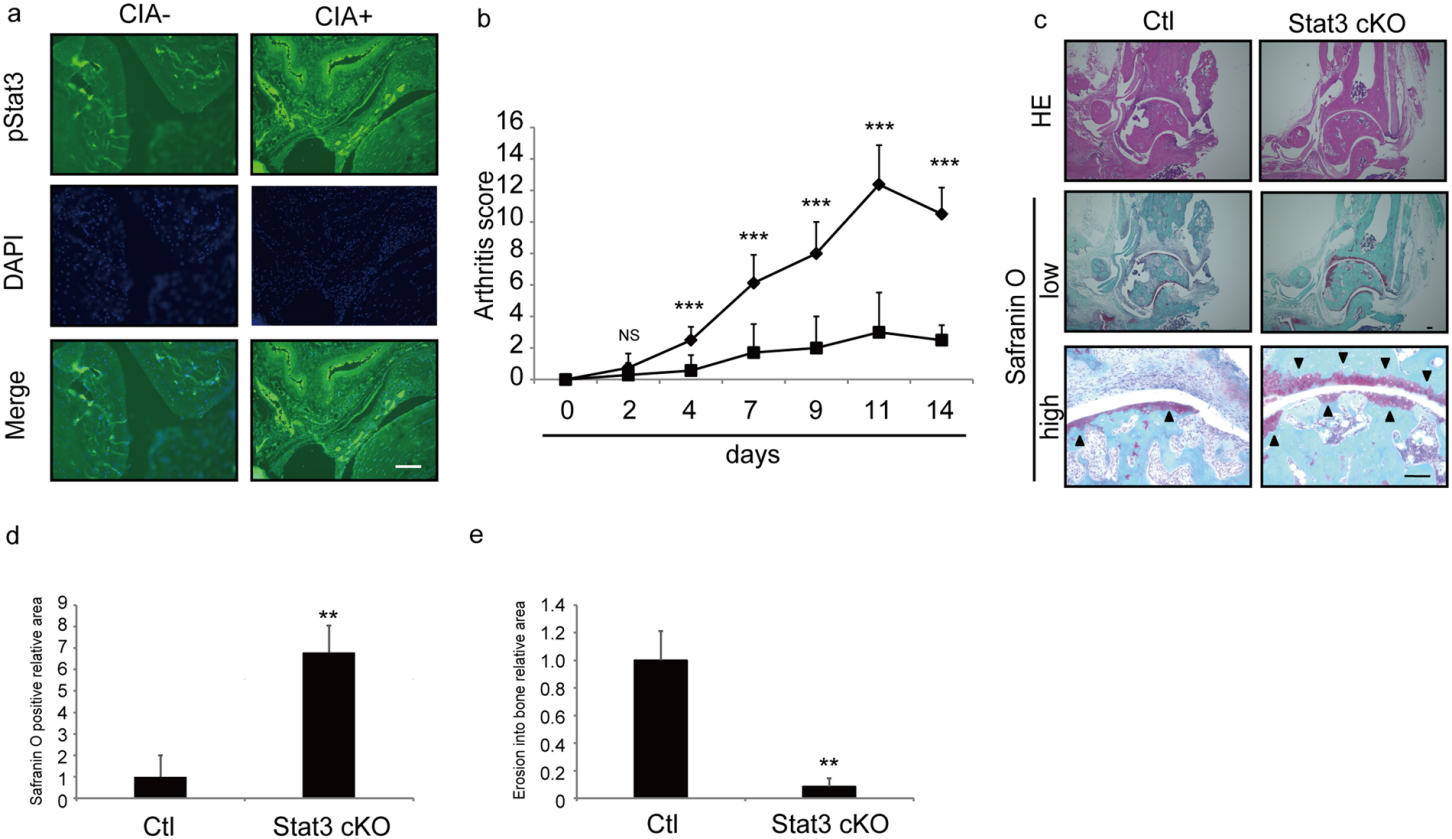
Stat3 is activated and required for arthritis development in CIA models. (**a–c**) 5-week-old wild-type DBA/1 J male mice were given an initial injection of type II collagen with CFA on day -21, and arthritis was induced with a second injection on day 0. Specimens of ankle joints from control or CIA mice were subjected to immunofluorescence staining 14 days after the second injection for pStat3. Nuclei were visualized by DAPI. Bar, 100 µm (**a**). CIA was induced in 5-week-old control (Ctl) or Stat3 cKO mice as above, and mice were co-administered PolyIpolyC (1.25 µg/kg/day) IP on days -21, -20, -19, -14 and -7 before the second type II collagen with CFA injection. An arthritis score was calculated at indicated time points after the second injection (**b**) and tissue specimens of ankles from Ctl or Stat3 cKO mice were stained 14 days after the second injection with hematoxylin eosin (upper panels) or safranin O and methyl green (middle and lower panels, low = low magnification, high = high magnification) (**c**). Data in (**b**) represent mean arthritis score ± SD (*n* = 8 for control, *n* = 7 for Stat3 cKO mice, ***P < 0.001, NS not significant). Data in (**d**) and (**e**) represent mean relative areas ± SD of safranin O-positive (**d**) and erosion into bone (**e**), respectively (*n* = 3 for control, *n* = 3 for Stat3 cKO mice, **P < 0.01). Arrowheads indicate safranin O-positive areas. Bar in (**c**) 100 µm.

We previously demonstrated that Stat3 regulates chronic inflammation^6^. Thus to investigate potential Stat3 activation in joint inflammation we employed CIA models. Using immunohistochemical analysis (Fig. 1a) we detected expression of activated (phosphorylated) pStat3 in synovium and subchondral bones in the joints of CIA model mice 14 days after the second type II collagen injection.

To assess Stat3 function in joints of CIA mice, we generated Stat3 conditional knockout mice in adults by crossing Mx Cre and Stat3 flox mice to yield Mx Cre/*Stat3* ^*flox/flox*^ mice (Stat3 cKO). We then injected Stat3 cKO and control (*Stat3* ^*flox/flox*^) mice with pIpC at five weeks of age to delete Stat3 and then established CIA models by injection of an emulsion of type II collagen (see Methods) (Fig. 1b–e). Others have reported that Stat3 deletion in macrophages and neutrophils results in macrophage hyperactivation, as determined by elevated *TNFα* expression in response to LPS^15^. Indeed, we found that Stat3 cKO macrophages showed higher *TNFα* expression after LPS stimulation than did control cells (Fig. S1a). However, joint inflammation, as assessed by an arthritis score, was significantly decreased in Stat3 cKO relative to control mice at the indicated time points (Fig. 1b). Stat3 deletion in macrophages and neutrophils reportedly promotes enterocolitis development^15^. However, our histological analysis revealed that joint destruction seen in control mice, as determined by loss of safranin O-positive articular cartilage and pannus infiltrate, was significantly blocked in Stat3 cKO mice at day 14 (Fig. 1c,d and e). Moreover, enterocolitis or the presence of F4/80-positive macrophage infiltrate in the colon was not evident in Stat3 cKO mice at this time point (Fig. S1b and c).

**Figure 2:**
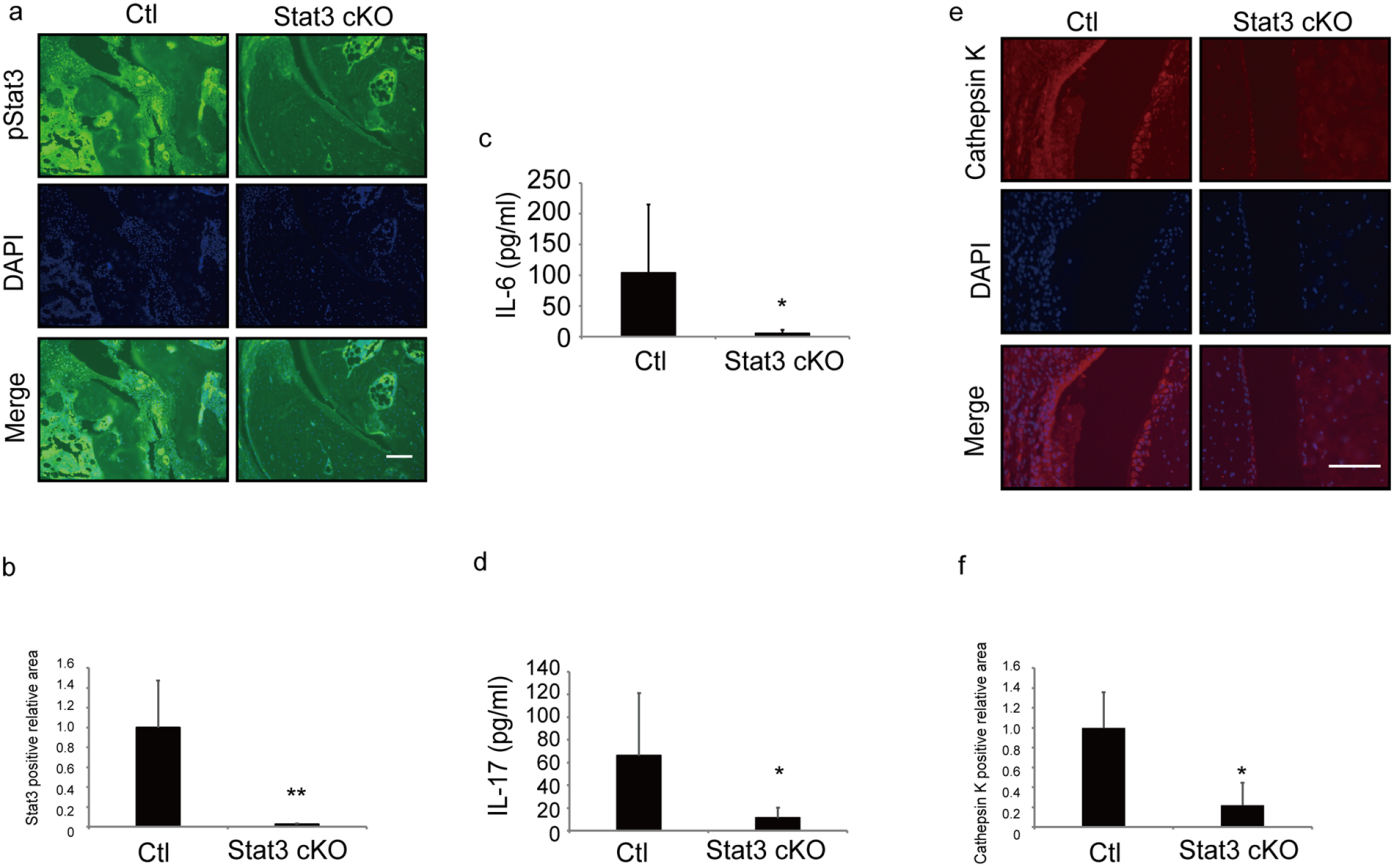
Stat3 is required for inflammation and osteoclast activation in CIA models. (**a–f**) CIA was induced by collagen injection in 5-week-old control (Ctl) or Stat3 cKO mice, and mice were administered PolyIpolyC (1.25 µg/kg/day) IP on days -21, -20, -19, -14 and -7 before a second type II collagen with CFA injection. Serum IL-6 (**c**) and IL-17 (**d**) protein levels in Ctl or Stat3 cKO mice were assessed by ELISA 14 days after the second injection. Specimens of ankle joint tissues from CIA Ctl or Stat3 cKO mice were subjected to immunofluorescence staining using pStat3 (**a**) or Cathepsin K (**e**) antibodies. Nuclei were stained with DAPI. Bar, 100 µm. Data in (**c**) represent mean IL-6 (pg/ml) ± SD (*n* = 8 for control, *n* = 7 for Stat3 cKO mice, *P < 0.05). Data in (**d**) represent mean and IL-17 (pg/ml) ± SD (*n* = 5 for control, *n* = 6 for Stat3 cKO mice, *P < 0.05). Data in (**b**) and (**f**) represent mean relative areas ± SD of Stat3-positive (**b**) and Cathepsin K-positive (**f**) cells, respectively (*n* = 3 for control, *n* = 3 for Stat3 cKO mice, *P < 0.05).

Immunohistochemical analysis 14 days after the second type II collagen injection confirmed that Stat3 was successfully and significantly deleted in Stat3 cKO mice (Fig. 2a and b). We also found that serum levels of the inflammatory cytokines IL-6 and IL-17 were significantly lower in Stat3 cKO relative to control mice (Fig. 2c and d) and detected Cathepsin K-positive osteoclast formation in control CIA mice but not in Stat3 cKO mice even under CIA conditions (Fig. 2e and f). These findings show that Stat3 loss antagonizes arthritic inflammation *in vivo*.

### Identification of Stat3 inhibitors

To search for Stat3 inhibitors, we undertook a Stat3 reporter assay to screen candidate drugs (Fig. S2). HeLa cells were stably transfected with a reporter consisting of four tandem Stat3 response elements linked to a luciferase gene and treated with the Stat3 activator Oncostatin M (OSM), and then their lysates were monitored for luciferase activity after eight hours of stimulation (Fig. S2). Ninety-six drugs approved to treat various human diseases were selected as previously described^16^, and then screened for potential inhibition of OSM-stimulated Stat3 reporter activity. Table 1 shows the top ten drugs from the screen identified as candidate Stat3 inhibitors. These drugs are currently used as histamine H1 receptor blockers (Nos 1 and 2), NSAIDs (Nos 9 and 10), calcium channel blockers (Nos 11, 12, 15 and 71), a gastric mucosa protector (No. 16) and a proton pump inhibitor (No. 18) (Table 1).

**Table 1:** Potential Stat3 inhibitors.

Drug number	Drug	Effect
1	Ebastine	Histamine H1 receptor blocker
2	Cetirizine hydrochloride	Histamine H1 receptor blocker
9	Meloxicam	NSAID
10	Loxoprofen sodium hydrate	NSAID
11	Nicardipine hydrochloride	Calcium channel blocker
12	Amlodipine besylate	Calcium channel blocker
15	Flavoxate hydrochloride	Calcium channel blocker
16	Sofalcone	Gastric mucosa protector
18	Lansoprazole	Proton pump inhibitor
71	Benidipine	Calcium channel blocker

**Figure 3:**
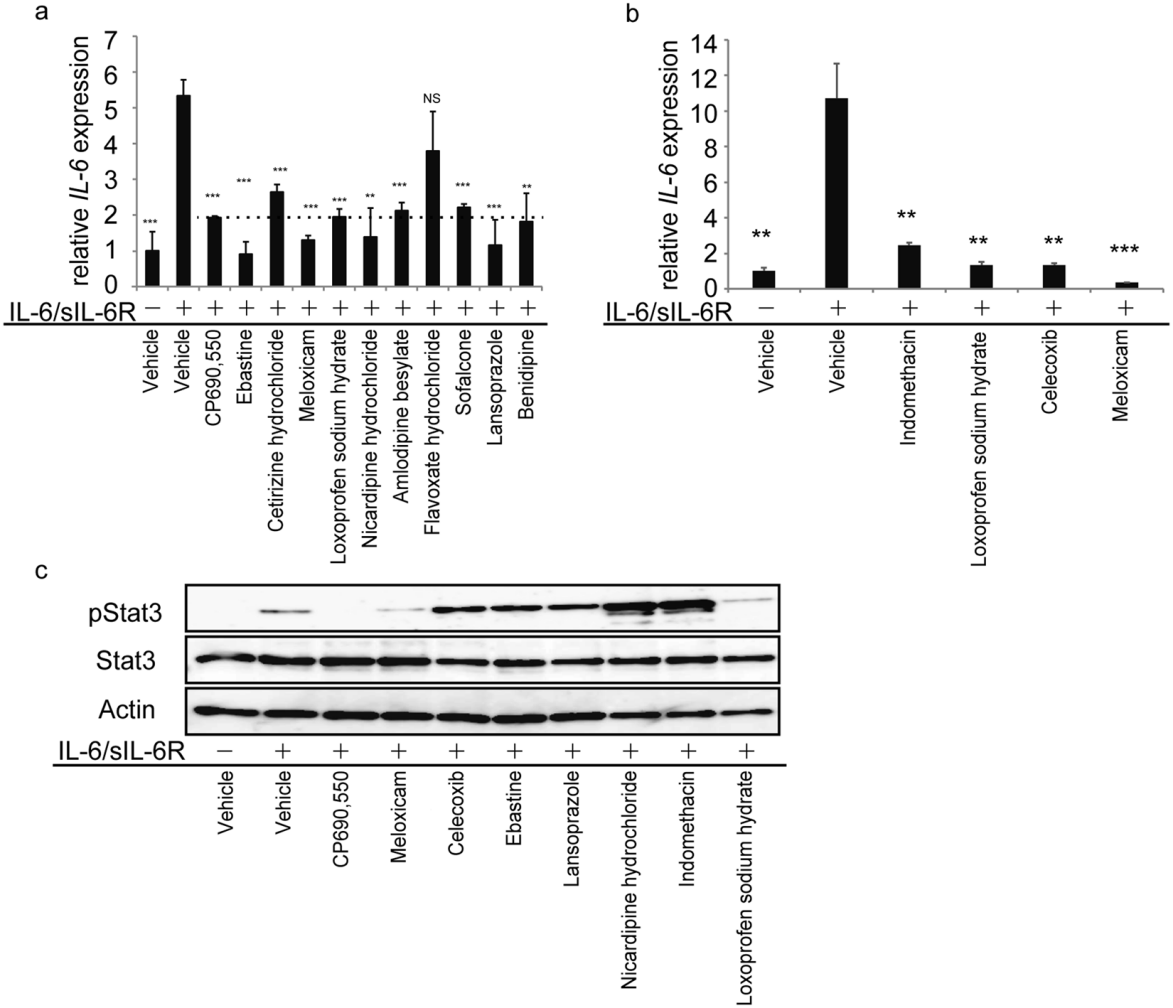
Drug screen for Stat3 inhibitors. (**a** and **b**) Total RNA was prepared from NIH3T3 cells treated with or without IL-6 (10 ng/ml) plus sIL-6R (100 ng/ml) in the presence or absence of indicated drugs (1 μM each) for 8 h, and *IL-6* mRNA expression relative to *β actin* was then analyzed by quantitative real-time PCR. Shown is mean *IL-6* expression relative to *β actin* ± SD (*n* = 3, *P < 0.05, **P < 0.01, ***P < 0.001, NS not significant, vs IL-6/sIL-6R + /vehicle). (**c**) NIH3T3 cells were treated with or without IL-6 (100 ng/ml) plus sIL-6R (100 ng/ml) in the presence or absence of indicated drugs (10 μM each) for 10 min and then whole-cell lysates were collected and immunoblotted to detect pStat3 or total Stat3. Actin served as an internal control. Representatives of at least two independent experiments are shown.

IL-6 expression is reportedly upregulated via an IL-6/Stat3-dependent positive feedback mechanism^17^. Thus, in an additional screen of the ten candidate drugs (Fig. 3), we stimulated NIH3T3 fibroblasts with IL-6 plus soluble IL-6 receptor (sIL-6R) in the presence or absence of each candidate drug for eight hours and assessed *IL-6* mRNA expression by realtime PCR (Fig. 3a)). CP690,550, which blocks Stat3 activation^6^, served as a positive control. Nine of ten drugs tested inhibited IL-6/sIL-6R-induced *IL-6* expression in this assay (Fig. 3a). Since the cyclooxygenase 2 (COX2) inhibitor meloxicam significantly inhibited *IL-6* mRNA expression in this last screen (Fig. 3a), we analyzed effects of other COX2 inhibitors and NSAIDs, such as indomethacin and celecoxib, as well as effects of loxoprofen sodium hydrate and meloxicam. We found that meloxicam was most potent in inhibiting self-induced *IL-6* mRNA expression by (Fig. 3b). From these results overall, we selected seven drugs (ebastine, meloxicam, lansoprazole, loxoprofen sodium hydrate, nicardipine hydrochloride, indomethacin and celecoxib) to test as Stat3 inhibitors in the next screen.

NIH3T3 cells were cultured with or without IL-6 plus sIL-6R in the presence or absence of indicated drugs for ten minutes to collect lysates for immunoblotting. Western analysis showed that Stat3 activation, as indicated by phosphorylation, was induced by IL-6 plus sIL-6R (Fig. 3c), while IL-6-dependent Stat3 activation was effectively blocked by meloxicam and loxoprofen sodium hydrate as well as the control CP690,550 (Fig. 3c), suggesting that both drugs inhibit Stat3 activation and phosphorylation. However, PDGFbb-stimulated activation of p38, Erk, Jnk and Akt in NIH3T3 cells was not blocked by either meloxicam or CP690,550 (Fig. S3). We conclude that Stat3 activation is specifically inhibited by meloxicam and CP690,550.

### Stat3 inhibitors effectively block joint inflammation and destruction in CIA models

**Figure 4:**
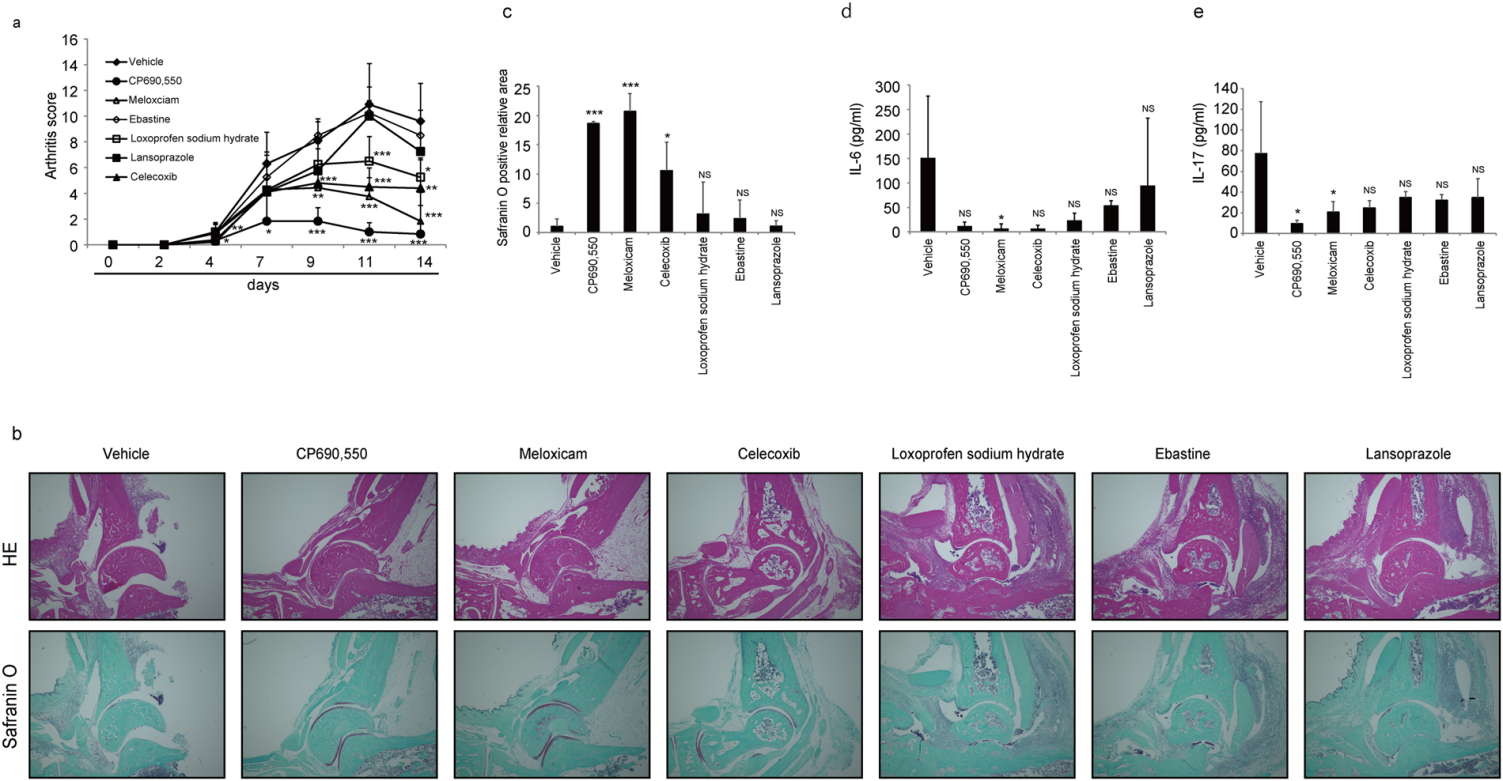
Effect of Stat3 inhibitors on arthritis development in CIA models. (**a**–**c**) 5-week-old wild-type DBA/1J male mice were initially injected with type II collagen with CFA on day -21, and arthritis was induced by a second injection on day 0. Indicated drugs (each 15 mg/kg/day) were administered IP once a day for 2 weeks starting at day 0. An arthritis score was evaluated at indicated time points after the second injection (**a**). Histological analysis was performed using hematoxylin eosin staining (HE, upper panels) or safranin O and methyl green (lower panels) staining of ankle joints from mice treated with indicated drugs for two weeks (**b**), and the safranin O-positive area was quantified (**c**). Serum IL-6 (**d**) and IL-17 (**e**) protein levels were also examined by ELISA in these mice treated with indicated drugs for two weeks. Data represent mean arthritis score (**a**), IL-6 (d) or IL-17 levels (**e**) ± SD (a, vehicle *n* = 10, CP690,550 *n* = 8, meloxicam *n* = 7, celexib *n* = 5, ebastine, loxoprofen sodium hydrate or lansoprazole *n* = 4 each; d, vehicle or meloxicam *n* = 5 each, CP690,550, celexib, loxoprofen sodium hydrate or lansoprazole *n* = 4 each, ebastine *n* = 3; e, vehicle or CP690,550 *n* = 7 each, meloxicam or loxoprofen sodium hydrate n = 4 each, celecoxib, ebastine or lansoprazole *n* = 3 each; *P < 0.05, **P < 0.01, ***P < 0.001, NS not significant, vs vehicle). Bar, 100 µm. Data in (**c**) represent mean relative safranin O-positive areas ± SD (*n* = 3 each; *P < 0.05, ***P < 0.001, NS not significant, vs vehicle). Representatives of at least two independent experiments are shown.

We next assessed effects of selected drugs on mouse CIA models established by injection of emulsions of type II collagen (See Methods). Once models were established, mice were administered the indicated drugs at day of the second collagen injection, and effects on joint inflammation were evaluated by an arthritis score (Fig. 4a). Among drugs tested, the COX2 inhibitors meloxicam and celecoxib and the NSAID loxoprofen sodium hydrate significantly inhibited joint inflammation (Fig. 4a). Safranin-O staining of the ankle joint sections from CIA mice after two weeks of the second collagen injection indicated that erosion of articular cartilage was effectively and significantly blocked by meloxicam treatment (Fig. 4b and c). Serum IL-6 and IL-17 levels in CIA mice were also significantly inhibited by meloxicam (Fig. 4d and e). Serum levels of IL-6 and IL-17 were also inhibited by celecoxib, loxoprofen sodium hydrate and ebastine (Fig. 4d and e), although those changes were not statistically significant. Thus, we found meloxicam to be the most potent inhibitor in terms of arthritis score, affect on serum IL-6 and IL17 levels and cartilage erosion in CIA mice.

**Figure 5:**
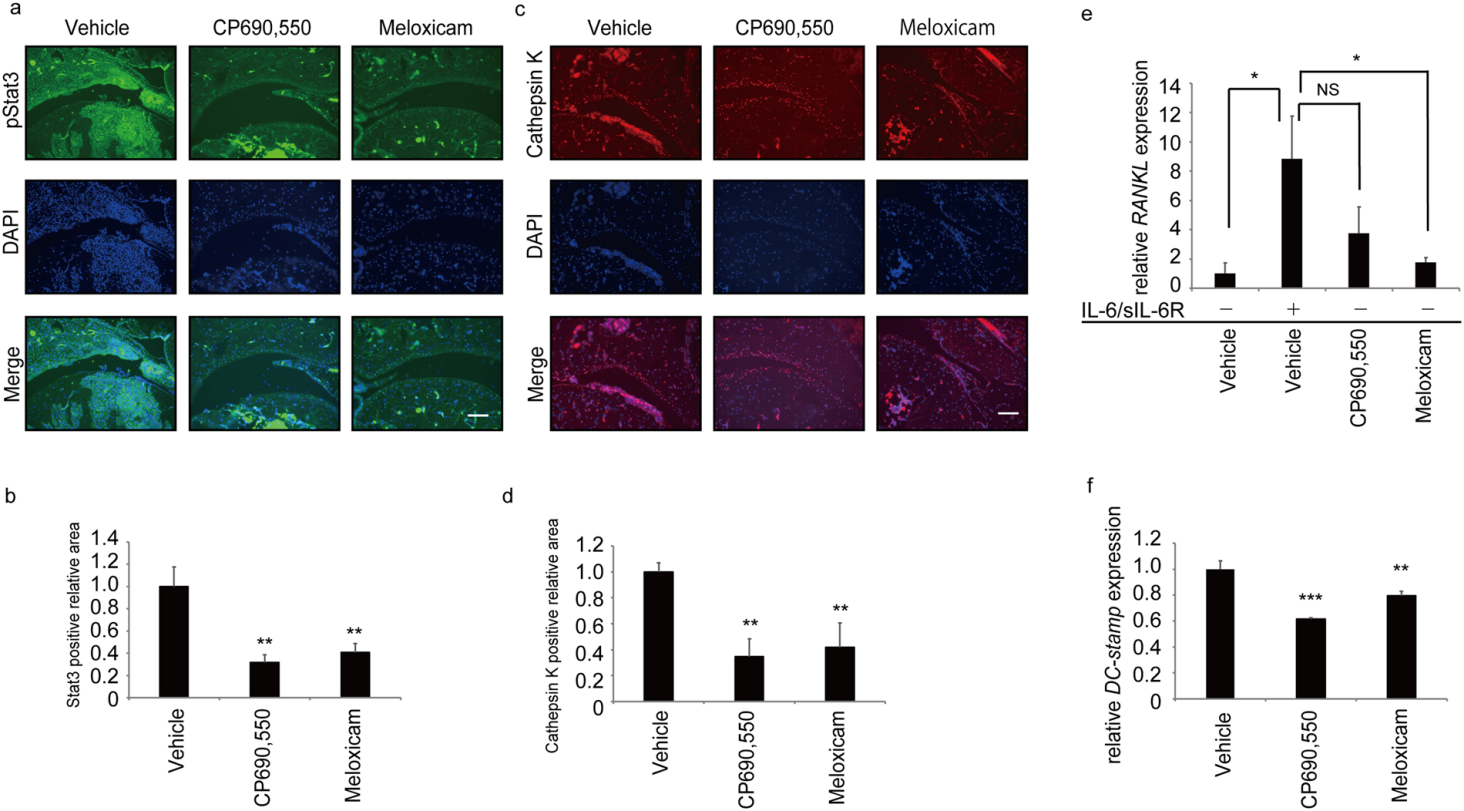
Effect of Stat3 inhibitors on Stat3 activation and osteoclast formation in CIA models. (**a**–**d**) Arthritis was induced in 5-week-old wild-type DBA/1 J male mice by injection of type II collagen and CFA on day -21 followed by the second injection on day 0. Vehicle, CP690550 or meloxicam (each 15 mg/kg/day) was administered IP once a day for 2 weeks starting at day 0. Specimens of ankle joints derived from CIA mice administered indicated drugs were subjected to immunofluorescence staining for pStat3 (**a**) or Cathepsin K 14 days after the second injection (**c**). Nuclei were visualized by DAPI. Bar, 100 µm. Data in (**b**) and (**d**) represent mean relative areas ± SD of Stat3-positive (**b**) and Cathespsin K-positive (**d**) cells, respectively (*n* = 3 each, **P < 0.01). (**e**) Total RNA was prepared from NIH3T3 cells treated 8 h with or without IL-6 (100 ng/ml) plus sIL-6R (100 ng/ml) in the presence or absence of vehicle, CP690550 or Meloxicam (1 μM). *RANKL* expression was then analyzed by realtime PCR. Data represents mean *RANKL* expression relative to *β actin* ± SD (*n* = 3, *P < 0.05, NS not significant). (**f**) M-CSF-dependent wild-type bone marrow cells (1 × 10^5^ cells per well) were cultured with osteoblastic MC3T3E1 cells (1 × 10^4^ cells per well) plus IL-6 (100 ng/ml) and sIL-6R (100 ng/ml) in the presence or absence of indicated drugs (1 μM each). Eight days later, mRNA was harvested for osteoclastic marker analysis. Representatives of at least two independent experiments are shown.

Finally, we asked whether meloxicam inhibits Stat3 activation in CIA mouse joints by immunohistochemical analysis two weeks after the second collagen injection (Fig. 5a). We found that Stat3 activation, as indicated by phosphorylation (pStat3), was effectively blocked by meloxicam in CIA mice (Fig. 5a and b). Formation of Cathepsin K-positive osteoclasts was also inhibited by meloxicam treatment of CIA mice (Fig. 5c and d). Furthermore, *RANKL* expression by NIH3T3 cells as induced by IL-6 and sIL-6R for eight hours *in vitro* was also significantly inhibited by meloxicam (Fig. 5e). Indeed, *in vitro* osteoclast formation as assessed by expression of an osteoclastic marker, *dendritic cell specific transmembrane protein* (*DC-STAMP*), induced by co-cultivation of osteoblastic MC3T3-E1 and bone marrow derived osteoclast progenitor cells in the presence of IL-6 and sIL-6R was significantly inhibited by meloxicam (Fig. 5f). Overall, our data show that meloxicam acts as a Stat3 inhibitor and can inhibit both joint inflammation and osteoclast formation in inflammatory arthritis.

## Discussion

RA is a disease of chronic joint inflammation and destruction^18^, in which inflammation is promoted by elevated levels of inflammatory cytokines such as IL-6, whereas joint destruction occurs via osteoclast activity^19^. Thus, effective treatment for RA patients would require inhibition of induction of both inflammatory cytokines and osteoclast activity. In this study, we demonstrated that Stat3 is a key regulator of both RA-related events and that inhibiting its activity is sufficient to block both inflammation and osteoclast activities in joints in a mouse model of arthritis. We then undertook a screen to identify Stat3-inhibiting drugs among existing drugs and found that meloxicam, a COX2 inhibitor, could block both inflammation and joint erosion in those model mice. Our screen represents a useful method to identify RA treating agents.

Current RA treatment often involves treatment with biologic agents, some of which have produced dramatic therapeutic effects^20^; however, biologic agents are expensive and cost is a concern for many patients. One of our goals was to identify less expensive reagents for RA therapy among previously tested drugs as a way of launching drug(s) for RA therapy in a more timely and less expensive manner.

Candidate Stat3 inhibitors identified here were meloxicam and celecoxib, both COX2 inhibitors, the NSAIDs indomethacin and loxoprofen sodium hydrate, the histamine H1 receptor blocker ebastine, the proton pump inhibitor lansoprazole, and the calcium channel blocker nicardipine. Some of these inhibited Stat3 activation induced by IL-6 *in vitro* but were not effective in the CIA model. How these reagents inhibit Stat3 activity in other contexts is an issue for future study.

Meloxicam is a COX2-selective NSAID^21^. Both COX1 and 2 function in the arachidonic acid cascade to produce prostaglandin E2 (PGE2), a physiologically active inflammatory substance, and blocking this cascade prevents inflammatory pain and fever. In CIA models COX2 inhibitors reportedly block expression of fibroblast-activating prostaglandin^22, 23^. Indeed, we found that PGE2 production by synovial fibroblasts in CIA models was effectively inhibited by meloxicam treatment (Fig. S4), an effect that likely antagonizes joint inflammation rather than blocking IL-6 and IL-17. COX1 is expressed in normal tissues and play roles in protecting the gastric mucosa from gastric acids, while COX2 is induced in inflammatory settings^21^. Blocking both COX1 and 2 causes gastric mucosal damage leading to gastric ulcer. Thus, COX2-selective agents are more useful in inhibiting inflammation without gastric mucosal damage. We showed that indomethacin and celecoxib do not inhibit pSTAT3 but effectively decrease *IL-6* mRNA expression. Since both drugs are effective in inhibiting inflammation in CIA^24^, these drugs act through general anti-inflammatory mechanisms, not by inhibiting Stat3. Our study demonstrates that meloxicam acts as a Stat3 inhibitor by inhibiting inflammation and osteoclast activation, and may represent a safer treatment option than Cox inhibitors.

Stat3 is required for embryonic development, and Stat3 global knockout mice exhibit early embryonic lethality. However, we did not observe lethality following global inhibition of Stat3 in adults, indicating that Stat3 can potentially be targeted in adults. Macrophage- neutrophil-specific Stat3 inhibition by LysMcre reportedly results in chronic enterocolitis^15^, and *Stat3* mutations in humans reportedly cause hyper IgE syndrome^25^. However, we did not detect apparent enterocolitis in our Stat3 cKO mice, nor have elevated IgE levels or enterocolitis been reported in patients administered anti-Stat3 reagents. Stat3 activity also reportedly plays a role in tumorigenesis^26–29^, and Stat3 inhibitors are under development as chemotherapeutic agents^30–34^.

Taken together, our data demonstrate that Stat3 could serve as a therapeutic target to treat RA. We also identify Stat3 inhibitors that could be used in approaches to implement less expensive drug treatments for RA therapy.

## Methods

### Mice

C57B6 and DBA/1 J mice were purchased from Sankyo Labo Service (Tokyo, Japan). Animals were maintained under specific pathogen-free conditions in animal facilities certified by the Keio University animal care committee. Animal protocols were approved by that committee. All animal experiments were carried out in accordance with the Guidelines of the Keio University animal care committee.

### CIA model

The experimental CIA model was generated in 5-week-old male mice by injecting 200 µl of emulsion containing 100 µg of type II collagen (CII) (Collagen Research Center, Tokyo, Japan) intradermally at the base of the tail. The emulsion was composed of 1 mg/ml bovine CII dissolved in PBS and an equal volume of complete Freund’s adjuvant (CFA, Difco, Detroit, MI, USA). Three weeks later, a second immunization of CII and CFA was administered. Clinical symptoms of arthritis were evaluated visually in each limb and graded on a scale of 0–4; 0, no erythema or swelling; 0.5, swelling of one or more digits; 1, erythema and mild swelling of the ankle joint; 2, mild erythema and swelling involving the entire paw; 3, erythema and moderate swelling involving the entire paw and 4, erythema and severe swelling involving the entire paw. The clinical score for each mouse that we term the “arthritis score” was the sum of scores for all four limbs (maximum score 16).

### Chemicals, drugs and reagents

CP-690550 was purchased from Selleck Chemicals (Houston, TX, USA). Human oncostatin M (OSM), mouse IL-6 and mouse sIL-6Ra were purchased from R&D systems (Minneapolis, MN, USA). Ebastine, cetirizine hydorochloride, meloxicam, loxoprofen sodium hydrate, nicardipine hydrochloride, amlodipine besylate, flavoxate hydrochloride, lansoprazole, indometacin and celecoxib were purchased from Tokyo Chemical Industry (Tokyo, Japan). Sofalcone and benidipine hydrochloride were purchased from Wako Pure Chemical Industries (Osaka, Japan).

### Cell culture

NIH3T3 and HeLa cells were maintained in DMEM (Sigma–Aldrich Co.) containing 10% fetal bovine serum (FBS) with penicillin G and streptomycin.

To assess osteoclast formation *in vitro*, bone marrow cells isolated from wild-type mouse femurs and tibias were cultured for 72 h in MEM (Sigma-Aldrich Co.) containing 10% (vol/vol) heat-inactivated FBS (JRH Biosciences, KS, USA) and GlutaMax (Invitrogen Corp.) supplemented with M-CSF (50 ng/mL, Kyowa Hakko Kirin Co.). Subsequently, adherent cells were collected and cultured in 96-well plates (1 × 10^5^ cells per well) with osteoblastic MC3T3E1 cells (1 × 10^4^ cells per well) in the presence or absence of IL-6 (100 ng/ml, R & D Systems) and sIL-6R (100 ng/ml, R & D Systems). Eight days later, mRNA was collected for analysis of osteoclastic markers, as described in Realtime PCR analysis.

To analyze macrophage/neutrophil function *in vitro*, bone marrow cells from wild-type or Stat3 cKO mouse femurs and tibias were cultured 72 h in MEM (Sigma-Aldrich Co.) containing 10% (vol/vol) heat-inactivated FBS (JRH Biosciences, KS, USA) and GlutaMax (Invitrogen Corp.) supplemented with M-CSF (50 ng/mL, Kyowa Hakko Kirin Co.) and IFN*β* 1a (100U/ml, R & D Systems). Adherent cells were then collected and cultured in 96-well plates (1 × 10^5^ cells per well) with M-CSF (50 ng/mL, Kyowa Hakko Kirin Co.) in the presence or absence of LPS (0ng/ml 2ng/ml 10ng/ml 50ng/ml, R & D Systems). After 24 hr of cultivation, mRNA was collected for analysis of *TNFα* expression as described in Realtime PCR analysis.

### Stat3 reporter assay in HeLa cells

HeLa cells were stably transfected with a Stat3-response element/luciferase reporter construct (Signosis, Santa Clara, CA, USA). The day before performing the reporter assay, cells were trypsinized, harvested and plated into each well of a 96-well plate with 5 × 10^4^ cells in 100 µl DMEM (Sigma-Aldrich Co.) containing 10% FBS (JRH Biosciences, KS, USA). Cells were then incubated in a humidified incubator at 37 °C with 5% CO2 overnight. Cells were then treated with or without 10 ng/ml Oncostatin M (OSM) in the presence or absence of drugs^16^ for 8 hours more for maximal Stat3 activation. We tested 1 μM concentrations of 96 drugs in the Stat3 activation assay. Luciferase assays were undertaken following the manufacturer’s instructions (Signosis, Santa Clara, CA, USA).

### Enzyme-linked immunosorbent assay (ELISA)

IL-6 ELISA assays were undertaken following the manufacturer’s instructions (R&D systems, Minneapolis, MN, USA).

### Realtime PCR analysis

Total RNAs were isolated from cultured cells using TRIzol reagent (Invitrogen Corp.), and cDNA synthesis was performed using oligo(dT) primers and reverse transcriptase (Wako Pure Chemicals Industries). Quantitative PCR was performed using SYBR Premix ExTaq II reagent and a DICE Thermal cycler (Takara Bio Inc.), and data was analyzed by that calibrator. *β-actin* (*Actb*) expression was analyzed as an internal control. Primers for realtime PCR were as follows.

*β-actin*-forward: 5′-TGAGAGGGAAATCGTGCGTGAC-3′

*β-actin*-reverse: 5′-AAGAAGGAAGGCTGGAAAAGAG-3′

*IL-6*-forward: 5′-GTCCTTAGCCACTCCTTCTG-3′

*IL-6*-reverse: 5′-CAAAGCCAGAGTCCTTCAGAG-3′

*RANKL*-forward: 5′-GCATCGCTCTGTTCCTGTACTTT-3′

*RANKL*-reverse: 5′-CGTTTTCATGGAGTCTCAGGATT-3′

*DC-STAMP*-forward: 5′-TCCTCCATGAACAAACAGTTCCAA-3′

*DC-STAMP*-reverse: 5′-AGACGTGGTTTAGGAATGCAGCTC-3′

*TNFα*-forward: 5′-CTTCTGTCTACTGAACTTCGGG-3′

*TNFα*-reverse: 5′-CAGGCTTGTCACTCGAATTTTG-3′

### Immunoblotting analysis

Whole-cell lysates were prepared from cultures using RIPA buffer (1% Tween 20, 0.1% SDS, 150 mM NaCl, 10 mM Tris-HCl (pH 7.4), 0.25 mM phenylmethylsulfonylfluoride, 10 μg/mL aprotinin, 10 μg/mL leupeptin, 1 mM Na3VO4, 5 mM NaF (Sigma-Aldrich Co.)). Equivalent amounts of protein were separated by SDS–PAGE and transferred to a PVDF membrane (EMD Millipore Corp.) Proteins were detected using the following antibodies (all purchased from Cell Signaling Technology, Inc., Beverly, MA, USA): anti-pSTAT3 (#9131), anti-STAT3 (#4904), anti-pp38 (#9211), anti-p38 (#9212), anti-pErk (#9106), anti-Erk (#9102), anti-pJnk (#9255), anti-Jnk (#9252), anti-pAkt (#4051) and anti-Akt (#9272). Anti-Actin antibody was purchased from Sigma-Aldrich Co., St Louis, MO.

### Histopathology and fluorescent immunohistochemistry

The lower ankle or gut tissue of CIA mice was fixed in 10% neutral-buffered formalin and embedded in paraffin, and tissue blocks were cut into 4-μm sections. The lower ankle was decalcified in 10% EDTA, pH7.4, before embedding. Hematoxylin and eosin (H&E) or safranin-O staining was performed according to standard procedures, and then safranin-O positivity or pannus infiltrate area was measured using a BioRevo microscope and corresponding software (Keyence, Tokyo, Japan). For each fluorescent immunohistochemistry assay, sections were subjected to microwave treatment for 10 min in 10 mM citrate buffer solution (pH 6.0) for antigen retrieval. After blocking with 3% BSA in PBS for 1 h, sections were stained for 6 h with rabbit anti-mouse pSTAT3 (1:100 dilution; Cell Signaling Techniques, Inc.) or rabbit anti-mouse Cathepsin K (1:100 dilution; Abcam) at 4 °C. After washing in TBST, sections were stained with Alexa Fluor 488-conjugated goat anti-rabbit IgG (1:100 dilution; Invitrogen, for pStat3) or Alexa Fluor 546-conjugated goat anti-rabbit IgG (1:100 dilution; Invitrogen, for Cathepsin K) for 1 h at room temperature. Sections were also stained with rabbit anti-mouse PGE2 (1:100 dilution; Abcam) followed by Alexa Fluor 488-conjugated goat anti-rabbit IgG (1:100 dilution; Invitrogen) or Alexa Fluor 488 anti-mouse F4/80 antibody (BioLegend). DAPI (1:750; Wako Pure Chemicals Industries, Osaka, Japan) was used as a nuclear stain.

### Statistical analysis

Results are expressed as means ± s.d. Statistical significance of differences between groups was evaluated using Student’s t-test (*P < 0.05; **P < 0.01; ***P < 0.001; NS, not significant, throughout the paper).

## Electronic supplementary material

Supplementary Figures and Legends
