# Supplementary material for: Stat3 as a potential therapeutic target for rheumatoid arthritis
Source: Sci Rep. 2017 Sep 8;7:10965. doi: 10.1038/s41598-017-11233-w (PMC5591217; doi:10.1038/s41598-017-11233-w)
Supplement: Supplementary file 1 — Supplementary Figures and Legends [file 41598_2017_11233_MOESM1_ESM.pdf]

# Stat3 as a potential therapeutic target for rheumatoid arthritis

Takatsugu Oike[1], Yuiko Sato[1,2], Tami Kobayashi[1,3], Kana Miyamoto[1], Satoshi Nakamura[1], Yosuke Kaneko[1], Shu Kobayashi[1], Kengo Harato[1], Hideyuki Saya[4], Morio Matsumoto[1], Masaya Nakamura[1], Yasuo Niki[1] and Takeshi Miyamoto[1,2]

[1]Department of Orthopedic Surgery, [2]Department of Advanced Therapy for Musculoskeletal Disorders, [3]Department of Musculoskeletal Reconstruction and Regeneration Surgery, [4]Division of Gene Regulation, Institute for Advanced Medical Research, Keio University School of Medicine, 35 Shinano-machi, Shinjuku-ku, Tokyo 160-8582, Japan

## Supplementary Figure Legend

**Figure S1. *Stat3* deletion in macrophages elevates *TNFα* expression in response to LPS.**

(a) Bone marrow cells isolated from Stat3 cKO or control mice were cultured with M-CSF (50 ng/ml) for three days. IFN*β* 1a (100U/ml) was added to cultures to delete *Stat3*. Adherent M-CSF-dependent cells were then collected and further cultured with M-CSF (50 ng/ml) in the presence or absence of indicated concentrations of LPS for 1.5, 3 or 6 hours. mRNA was then collected and *TNFα* expression analyzed by realtime PCR. Data represent mean *TNFα* expression relative to *β-actin* ± SD (*, $p < 0.05$; **, $p < 0.01$; ***, $p < 0.001$; *ns*, not significant; $n = 3$). (b and c) Five-week-old wild-type or Stat3 cKO mice were initially injected with type II collagen with CFA, and a second injection was performed 21 days later to induce arthritis (collagen-induced arthritis, CIA). Fourteen days after the second injection, CIA mice were sacrificed and gut sections were stained with hematoxylin eosin (HE, b) or Alexa488-conjugated anti-F4/80 antibody. Nuclei were stained with DAPI (c). Bar, 100 µm.

**Figure S2. Drug screen for inhibitors of Stat3.**

Ninety-six existing drugs were screened in the presence of Oncostatin M (10 ng/ml), a Stat3 activator, as potential Stat3 inhibitors using a Stat3-response element/luciferase reporter assay. The dotted line indicates a cut-off value for Stat3-inhibition. CP690,550 was used a positive control as a Stat3 inhibitor.

**Figure S3. Stat3 is specifically inhibited by meloxicam.**

NIH3T3 cells were cultured with or without PDGFbb (100 ng/ml) in the presence or absence of indicated drugs (10 μM each) for 10 min. Whole-cell lysates were then collected and immunoblotted to detect pp38, pErk, pJnk, pAkt, p38, Erk, Jnk or Akt. Actin served as an internal control. Representatives of at least two independent experiments are shown.

**Figure 4. Meloxicam treatment inhibits Prostaglandin 2 expression.**

Five-week-old wild-type DBA/1J male mice were injected with type II collagen plus CFA on day -21, and arthritis was induced by a second injection on day 0. Either CP690,550 or meloxicam (each 15mg/kg/day) was administered IP once a day for 2 weeks, starting at day 0. Specimens of ankle joints from control or CIA mice were subjected to immunofluorescence staining 14 days after the second injection to detect prostaglandin 2 (PGE2). Nuclei were visualized by DAPI. Bar, 100 μm.

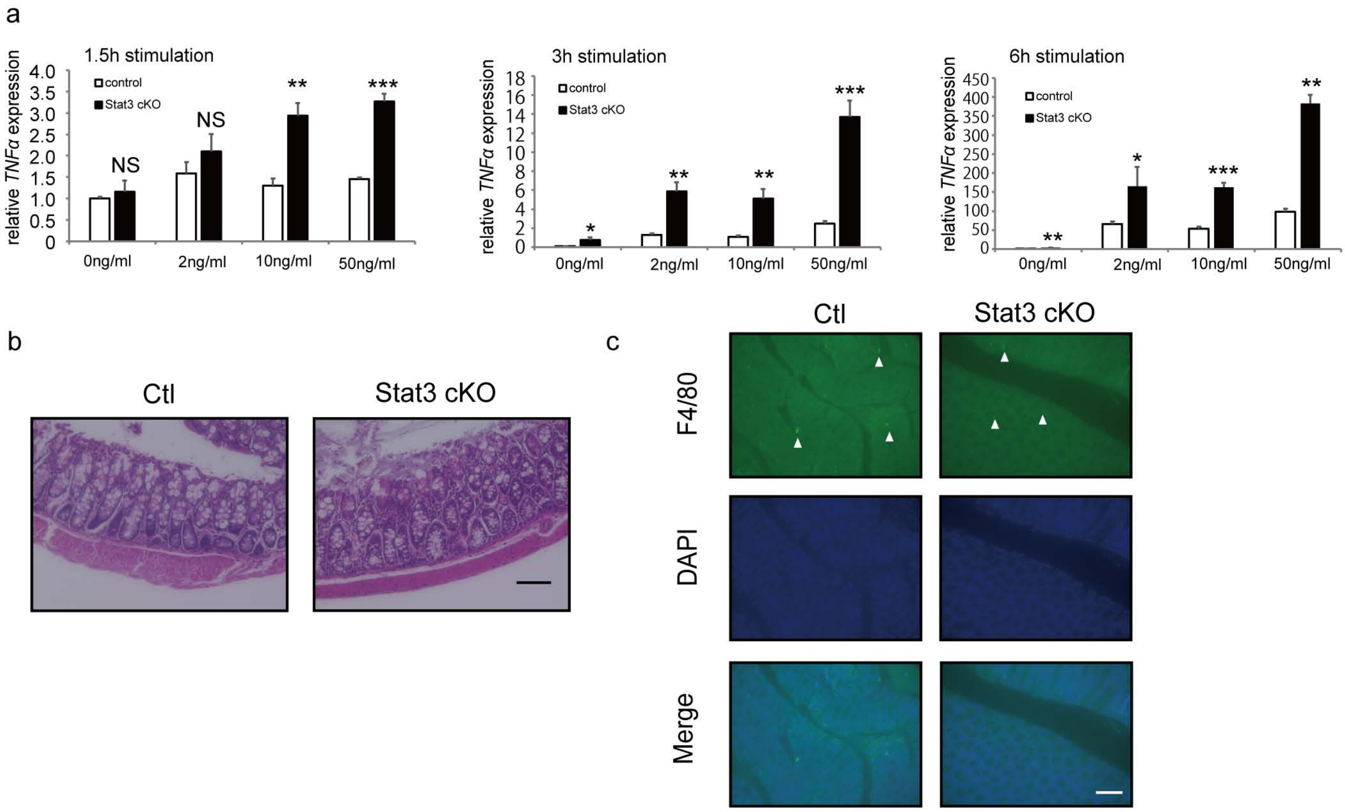


Figure S1.Oike T.et al.

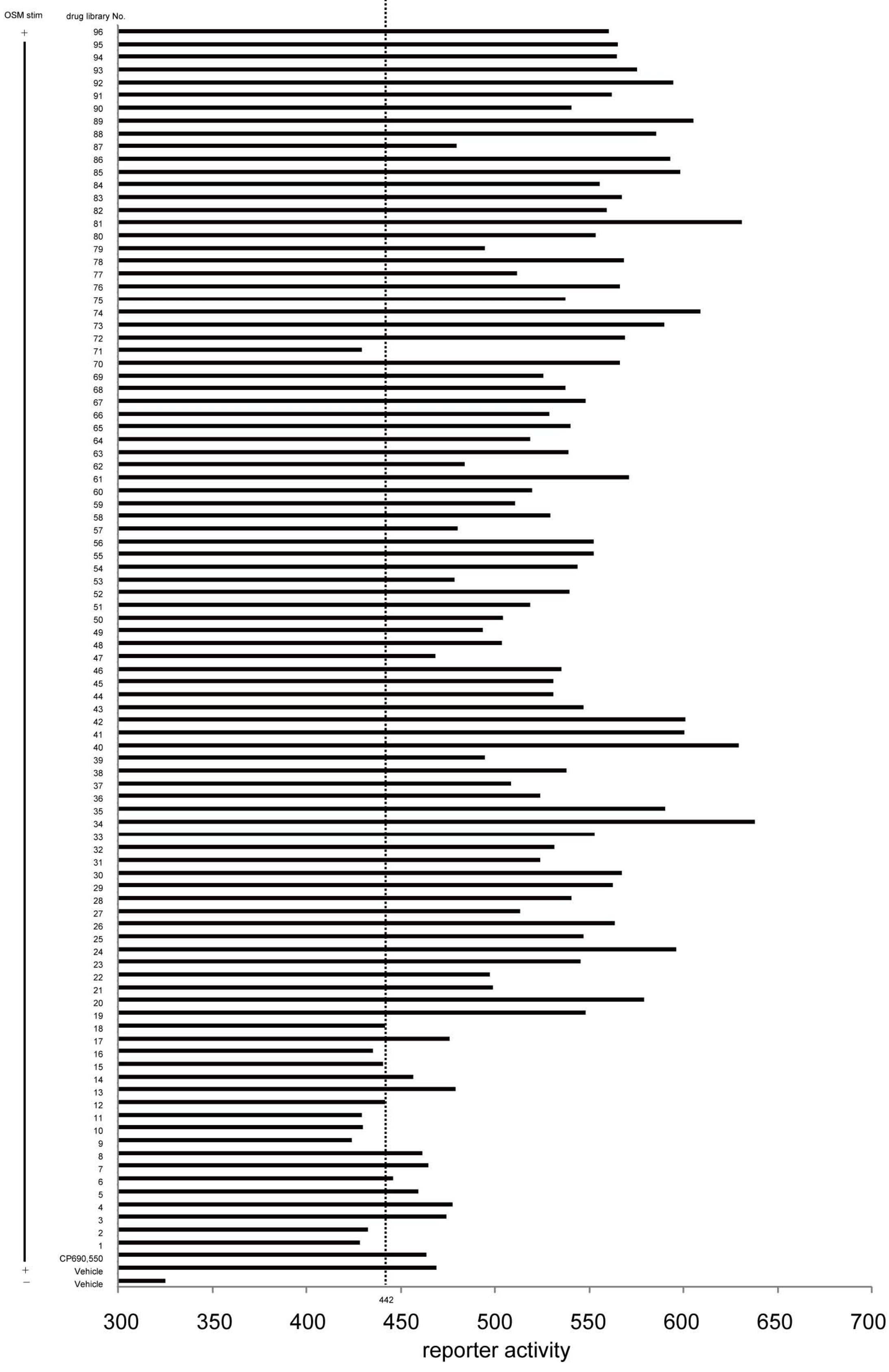


Figure S2.Oike T.et al.

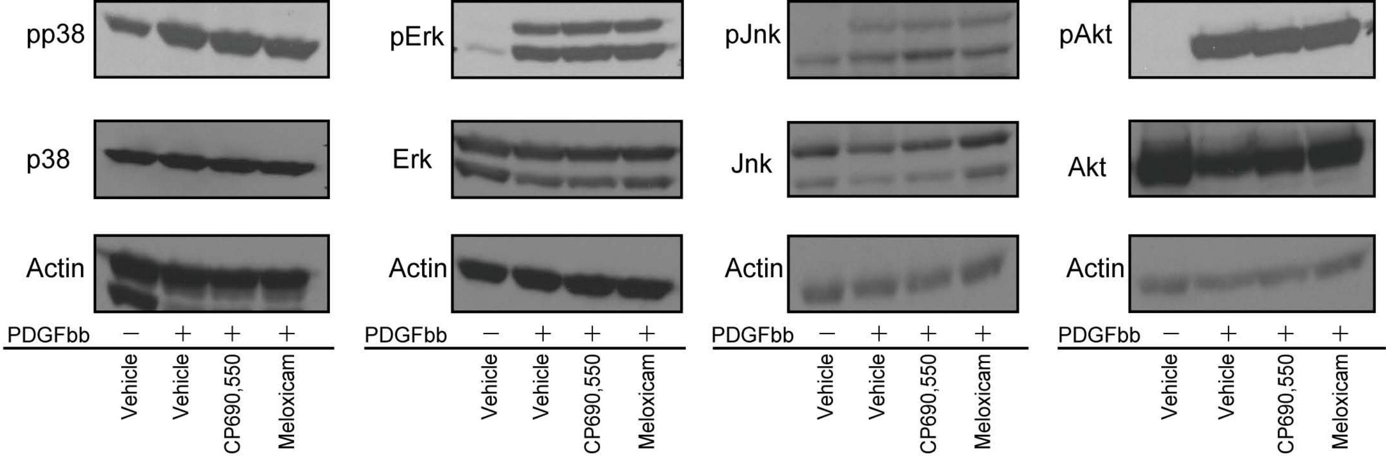


Figure S3.Oike T.et al.

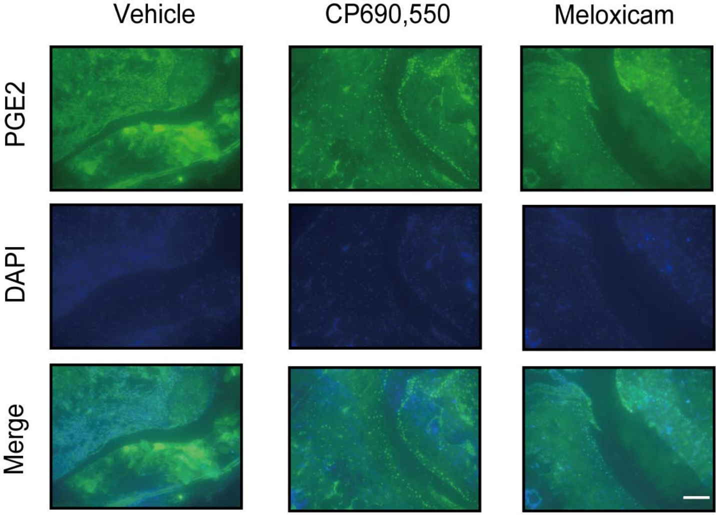


Figure S4.Oike T.et al.
